# Psychometric properties and invariance of an English self-efficacy scale for university students in Peru

**DOI:** 10.3389/fpsyg.2023.1187342

**Published:** 2023-06-15

**Authors:** Mariela Estela Mendoza-Torres, Wilter C. Morales-García, Liset Z. Sairitupa-Sanchez, Sandra B. Morales-García, Oriana Rivera-Lozada, Francisco E. Sucapuca-Sucapuca, Denis Frank Cunza-Aranzábal

**Affiliations:** ^1^Unidad de Educación, Escuela de Posgrado, Universidad Peruana Unión, Lima, Peru; ^2^Escuela de Medicina Humana, Facultad de Ciencias de la Salud, Universidad Peruana Unión, Lima, Peru; ^3^Facultad de Teología, Universidad Peruana Unión, Lima, Peru; ^4^Escuela de Posgrado, Universidad Peruana Unión, Lima, Peru; ^5^Escuela Profesional de Psicología, Facultad de Ciencias de la Salud, Universidad Peruana Unión, Lima, Peru; ^6^Facultad de Farmacia y Bioquímica, Universidad Científica del Sur, Lima, Peru; ^7^South American Center for Education and Research in Public Health, Universidad Norbert Wiener, Lima, Peru; ^8^Facultad de Ciencias Químicas, Universidad Nacional San Agustín de Arequipa, Arequipa, Peru; ^9^Faultad de Ciencias de la Salud, Universidad Peruana Unión, Tarapoto, Peru

**Keywords:** self-efficacy, English, students, psychometric properties, invariance

## Abstract

**Background:**

English is a global language used to communicate with members of the international community. Self-efficacy in learning English is an important factor that is influenced by perceived importance, interest, and beliefs about the ability to successfully perform tasks in English.

**Objective:**

The aim is to develop and validate a measurement instrument to assess English self-efficacy.

**Methods:**

A total of 453 students from different Peruvian universities participated, with ages ranging from 18 to 60 years (M = 23; SD = 6.18). Statistical techniques for latent variables were used and recommendations for educational and psychological tests were followed in its construction. The sample was divided into two groups to perform exploratory factor analysis (EFA) and confirmatory factor analysis (CFA).

**Results:**

The English Self-Efficacy Scale (ESS-P) is representative and relevant in terms of its item content (Aiken’s V > 0.70). Its internal structure is organized into three first order factors and a second order factor that are consistent with the theoretical proposal and was confirmed through CFA with excellent goodness-of-fit indices (χ^2^ = 1184.9, gl = 626, CFI = 0.92, TLI = 0.92, RMSEA = 0.06 y SRMR = 0.04). It also has adequate internal consistency in its three factors (Reading α/ω = 0.96), Oral Communication (α/ω = 0.95), and Writing (α/ω = 0.97) and the whole scale (α/ω = 0.98), is invariant with respect to sex, and has a conceptual relationship with variables such as academic self-efficacy and exam anxiety.

**Conclusion:**

The ESS-P is a measurement instrument with evidence of validity, factorial invariance, and good reliability of its scores. Therefore, it can be used in future studies in the academic context.

## 1. Introduction

English is referred to as the global language due to its use in connecting and communicating with members of the international community (Botes et al., 2020). Nonetheless, the level of English proficiency as a foreign language in Peru is moderate, ranking 51st in Education First English Proficient Index (2022). The learning of this language for non-native speakers is not an easy process, being influenced by various motivational factors: perceived importance, interest, and self-efficacy (Bai et al., 2020). The latter constitutes a topic of research in the field of English as a Foreign Language (EFL).

Positive psychology has been a rapidly growing field in the last decade, focusing on how individuals can live optimally and reach their full potential through an approach based on empirical data and scientific methods (Seligman and Csikszentmihalyi, 2000; Seligman, 2004). Within the subfield of second language learning, positive psychology has been applied in various contexts and levels of identity, ranging from a general trait to a specific state (Mercer and MacIntyre, 2014; Gabryś-Barker and Gałajda, 2016; Mercer et al., 2018). Self-efficacy refers to the set of beliefs that individuals have about their abilities to perform activities necessary to achieve desired goals (Bandura, 1977). This construct is based on the Social Cognitive Theory (SCT; Bandura, 2008). In the educational field, the beliefs that students have about their own capabilities play an important role, not only in general but also in a specific area such as English, and specific items can be used to measure self-efficacy in that area (Bai et al., 2020). Therefore, English self-efficacy is defined as the belief that a person has about the effectiveness of their abilities to perform a task in English successfully (Wang et al., 2014).

In the context of second language learning, it has been found that self-efficacy in specific areas, such as reading, speaking, and listening comprehension, is related to positive self-concept variables and language competence (Chen et al., 2020). Currently, there are two approaches to the dimensional structure of English self-efficacy. The first approach has been divided into four categories (Wang et al., 2014; Wang and Bai, 2017; Ngoc Truong and Wang, 2019), these are: (a) Reading, which refers to the ability to comprehend written text in English, taking into account the purpose of reading, recognition of key words, identification of explicit and implicit main ideas, type of text, and manifestation of a critical position (Newton et al., 2018); (b) Speaking, which encompasses the ability to communicate orally in English spontaneously, asking questions to obtain information about a specific topic, as well as answering the questions of the interlocutor, accompanied by facial expressions, gestures, or body movements (Burns, 2019); (c) Writing, which involves writing a specific type of text in English in a coherent and cohesive manner, considering the purpose, recipient, type of language to use, and writing processes (planning, first draft, revision, and final draft; Hyland, 2019); and (d) Listening, which refers to the ability to understand what the English speaker communicates, identifying the content of the message (Gilakjani and Sabouri, 2016). On the other hand, according to the other approach, they are divided into three categories (Chauvin et al., 2020), these are: (a) media comprehension, related to understanding audio, radio, and video programs; (b) receptive and productive written communication, composed of reading and writing tasks; and (c) receptive and productive oral communication, related to the listening and speaking tasks that are found in social interaction (Chauvin et al., 2020).

Several instruments have been developed to evaluate English self-efficacy based on Wang’s qualitative study, with a sample of 4 boys aged 6 to 9 years (Wang, 2004), whose results could be more applicable to these age ranges, but they could lead to the omission of items most relevant for male and female adults. One of these instruments is the Questionnaire of English Self-Efficacy (QESE), which has a theoretical structure consisting of four dimensions and was developed from a qualitative approach using interviews, observations, and verbal protocols with Chinese elementary school English students in the United States (Wang, 2004). This instrument was later adapted in Korea with a sample of university students (Wang et al., 2013). This version was also evaluated with Chinese high school students aged 15 to 19 years (Wang and Bai, 2017) and university students in China, with modifications to some of its items (Wang et al., 2014). This same model was also applied to university students in Vietnam (Ngoc Truong and Wang, 2019). On the other hand, the Professional English Self-Efficacy Questionary (PESEQ) was designed from the same approach as the QESE and was applied to a professional French population (PESEQ) with the purpose of measuring English self-efficacy at the professional level. It showed a structure of three dimensions: media comprehension, receptive and productive written communication (reading and writing), and receptive and productive oral communication (listening and speaking; Chauvin et al., 2020). Both instruments have similar theoretical approaches with 4 and 3 dimensions, respectively, but have been applied to different populations: high school students, university students, and professionals. The ESS-P, proposed in the present study, has a structure similar to the scales mentioned above since it focuses on reading and writing, as well as oral communication which includes speaking and listening intrinsically; however, this scale differs from the previous ones because it introduces a set of new items developed from a different theoretical scope, and by its characteristics serves to evaluate self-efficacy for English in undergraduate and postgraduate students in general contexts, not exclusively for the academic environment such as QESE or only for the work context such as PESEQ.

Due to the fact that having a better proficiency in the English language requires students to have higher self-efficacy in performing various activities involving the language (Anam and Stracke, 2020), some researchers have found differences between men and women in terms of self-efficacy scores related to English achievement (Kutuk et al., 2022; Xu et al., 2022). For example, higher levels of self-efficacy for writing have been recorded for males compared to females (Ramos-Villagrasa et al., 2018); however, another study showed a stronger relationship between self-efficacy in writing and writing performance for female students than for male students (Pajares et al., 2001). A more recent study, on the other hand, revealed that the relationship between writing self-efficacy and writing performance was equal for both women and men, indicating that the significant differences in self-efficacy and writing performance in favor of female students were due more to women’s confidence in the stereotype that attributes higher self-efficacy for languages to them (Sun et al., 2021). Therefore, it is important to take into account measurement invariance, as it allows verifying whether members of different groups have the same understanding of the items on a scale. In this way, it ensures that people with the same level of a trait will give the same responses to the scale, regardless of their group affiliation (Milfont and Fischer, 2010). To make these comparisons, it is necessary that measurement invariance is met, as otherwise, it cannot be guaranteed that the indicators reflect the same construct in all groups and have the same meaning (Byrne, 2008; Dimitrov, 2010). Thus, it becomes necessary to compare self-efficacy for English between men and women (Byrne and Stewart, 2009), in order to estimate latent factors and make comparisons between individuals of both sexes, without varying the relevant properties of the scale, so as not to reach incorrect conclusions (García-Álvarez et al., 2022). If the measurement instruments have not shown invariance, the conclusions derived from the studies can be erroneous and biased toward one of the groups, without reflecting the true differences in the way people respond to the items (Byrne, 2008).

To develop adequate English self-efficacy, it is necessary to select cognitive and self-regulation strategies that motivate its development (Velasco-Zárate and Meza-Cano, 2020). English self-efficacy is positively related to academic self-efficacy, which indicates the reciprocal predictive capacity of both variables (Wang et al., 2018), and since academic self-efficacy has a negative association with exam anxiety (Preiss et al., 2006), a negative association between English self-efficacy and exam anxiety would also be expected. English self-efficacy also shows significant correlations with learning strategies (Montaño-González and Cancino, 2020) and academic achievement (Kitikanan and Sasimonton, 2017). Additionally, it has been shown that self-efficacy for academic writing in English can be improved through technological strategies (Velasco-Zárate and Meza-Cano, 2020).

In this sense, due to the lack of Spanish-speaking instruments that aim to measure self-efficacy, the purpose of this study is to develop an instrument that allows measuring English self-efficacy in university students, providing evidence in terms of content, internal structure, sex invariance, and relationship with other variables.

## 2. Methods

### 2.1. Design and participants

It is an instrumental research design (Ato et al., 2013). To calculate the sample size, we evaluated the size of the effect that includes the number of visible and hidden variables in the model, the expected effect (λ = 0.10), the desired level of statistical significance (α = 0.05) and the statistical power (1 − β = 0.90), which leads us to recommend a minimum sample of 199. A total of 418 students from 19 Peruvian universities, aged between 18 and 50 years (M = 21.8; SD = 3.94), participated in the study. The majority (51.7%) were women, attending public universities (66.5%), at a basic level of English language study (59.6%), majoring in Engineering, Industry, and Construction (42.1%), residing in the Ancash region (61.0%), and attending public universities (66.5). They were selected through non-probabilistic sampling (Table 1). Additionally, the sample was divided into two groups, with 230 cases randomly selected for the exploratory factor analysis (EFA) and 188 for the confirmatory factor analysis (CFA; Lloret-Segura et al., 2014).

**Table 1:** Detailed description of the study sample.

Characteristics		*n*	%
Sex	Women	216	51.7
	Men	202	48.3
Marital status	Single	396	94.7
	Married	15	3.6
	Cohabiting	6	1.4
	Divorced	1	0.2
Field of study	Engineering, industry, and construction	176	42.1
	Social sciences, commerce, and law	79	18.9
	Education	75	17.9
	Health sciences	65	15.6
	Natural sciences, exact sciences, and computing	11	2.6
	Agriculture and veterinary science	9	2.2
	Humanities and art	3	0.7
Location of university	Ancash	255	61.0
	Puno	2	0.5
	La Libertad	2	0.5
	Lima	1	0.2
	San Martín	38	9.1
	Cajamarca	34	8.1
	Cusco	1	0.2
	Piura	2	0.5
	Junín	53	12.7
	Loreto	30	7.2
Type of university	Public	278	66.5
	Private	140	33.5
English level	Basic	249	59.6
	Intermediate	151	36.1
	Advanced	18	4.3

### 2.2. Instruments

English Self-Efficacy Scale (ESS-P). The English Self-Efficacy Scale (ESS-P) was developed from a literature review (Gilakjani and Sabouri, 2016; Newton et al., 2018; Burns, 2019; Hyland, 2019), and aims to assess self-efficacy in English. Initially, it focused on four areas like previous instruments developed by other authors (Wang et al., 2014), but later three dimensions based on the main skills were proposed: reading (Newton et al., 2018), writing (Hyland, 2019), and speaking/listening (Gilakjani et al., 2016; Burns, 2019); which were combined into an oral communication dimension based on the criteria of Chauvin et al. (2020). Each of the items is measured on a 5-point Likert scale: 0 = I cannot do it at all, 1 = I cannot do it, 2 = Relatively sure I can do it, 3 = I can do it, 4 = Totally sure I can do it. Although it was originally recommended to use a 0 to 100 scale (Bandura, 2006), a subsequent review of self-efficacy scales has shown the frequent use of 5-response scales with satisfactory results (Betz, 2013), so a 5-response option was also chosen in the present study.

Single-Item Academic Self-Efficacy (IUAA; Dominguez-Lara et al., 2019). It is a measure of Academic Self-Efficacy, whose single question is, “How sure are you that you will be able to efficiently perform the tasks demanded of you in your academic life?,” which was organized under a five-point scale, from Not at all sure to Very sure.

Single-Item Test Anxiety (SITA; Dominguez-Lara, 2018). This is an instrument that evaluates exam anxiety through a single item: “During exams I feel a lot of tension” with 4 response alternatives: 1 = almost never, 2 = sometimes, 3 = frequently, and 4 = almost always.

### 2.3. Procedure

The research protocol was approved by the Research Ethics Committee of the Peruvian university with reference number CE-EPG-0000119. Contact was established with administrators of public and private universities throughout Peru. A pilot test was conducted through a Zoom meeting to identify problems in item writing and comprehension. Then, informed consent forms were sent through Google Forms, WhatsApp groups, and emails to students. Participants were allowed to leave the study at any time if they wished. Finally, the study was carried out following the ethical standards established in the Helsinki Declaration, including the protection of personal information privacy and confidentiality and the minimization of any impact on the physical, mental, and social health of participants.

### 2.4. Data analysis

To evaluate the content validity of the ESS-P, five university professors with experience in teaching English at the undergraduate and graduate level were asked to rate the relevance of the items and provide suggestions for improvement (Suárez-Alvarez et al., 2018). The responses were analyzed using the Aiken coefficient for dichotomous judge ratings (V = 1, *p* = 0.031; Aiken, 1985). A pilot test was conducted with 20 undergraduate and postgraduate students to verify the understanding of the items and to obtain the discriminate index and a preliminary reliability analysis (Muñiz and Fonseca-Pedrero, 2019).

Before starting the Exploratory Factor Analysis (EFA), certain conditions must be met, such as linear correlation in the item matrix and verification of common factors through the Kaiser-Meyer-Olkin (KMO) coefficient. High values of KMO indicate that the correlations between the items can be explained by other variables (Kaiser, 1974). In addition, the Barlett sphericality test was used to determine if the correlation matrix is an identity matrix, which means that the correlations are zero.

The Exploratory Factor Analysis (EFA) was performed using the minimum residual extraction method with oblimin rotation, considering factor loadings greater than 0.30 as a criterion for belonging to a factor (Nunnally and Bernstein, 1994) and the number of factors was determined through a parallel analysis. Subsequently, Confirmatory Factor Analysis (CFA) was chosen and estimated using the lavaan library in the RStudio interface. A confirmatory factor analysis was performed on the unifactorial scale using the MLR estimator, which is suitable for numerical variables and is robust to normality deviations in inference (Muthén and Muthén, 2017). Fit measures were used to evaluate the factor structure, such as the Confirmatory Fit Index and Tucker-Lewis Index (CFI and TLI ≥ 0.95; Schumacker and Lomax, 2016), the Root Mean Square Error of Approximation and the Standardized Root Mean Square Residuals (RMSEA and SRMSR ≤ 0.05; Kline, 2016). Factor loadings (λ) with values greater than 0.50 were considered adequate. In addition, the average extracted variance (AVE) was calculated and a value greater than 0.50 was considered appropriate, indicating that more than 50% of the variance of the construct is explained by its indicators (Fornell and Larcker, 1981).

In addition, reliability was evaluated through internal consistency using the Cronbach’s alpha coefficient (Cronbach, 1951) and McDonald’s Omega (Hayes and Coutts, 2020), which is suitable for factor models. The relationship with other convergent variables was also analyzed through correlations based on structural equation models between the scores of the English Self-Efficacy Scale (ESS-P), the single item of Academic self-efficacy, and Test Anxiety. An effect magnitude of r ≥ 0.20 was considered the minimum recommended, while r ≥ 0.50 was moderate and r ≥ 0.80 was strong.

To evaluate the consistency of the factors based on the sex of the participants, a series of increasingly strict hierarchical variance models were used. First, the configuration invariance (reference model) was evaluated, followed by metric invariance (equality of factor loads), scalar invariance (equality of factor loads and intercepts), and finally, strict invariance (equality of factor loads, intercepts, and residuals) was evaluated. To compare the models, a formal statistical test was first used, using the difference in the Confirmatory Fit Index (ΔCFI), where values lower than 0.010 provided evidence of model consistency between groups (Chen, 2007; Finch and French, 2018).

## 3. Results

### 3.1. Content validity analysis

To evaluate the content validity of the questionnaire, five experts in teaching English were recruited as judges. The 65 items were evaluated in terms of clarity, consistency, context, and relevance to the construct. The Aiken V Coefficient was used to analyze the results, and two items (6 and 26) were removed for not meeting the standards of clarity, consistency, context, and relevance to the construct (V = 0.80, *p* > 0.05).

### 3.2. Pilot test

The pilot test with 20 undergraduate and graduate students allowed for improvement of the instrument. Based on the collected data, the discrimination index of the items was evaluated using Pearson’s product–moment correlation between the values of each item and the sum total of the other items. It was found that item 6 had the lowest value and item 15 had the highest value (Min. = 0.429, Max. = 0.943), indicating that all values were appropriate (>0.2; Rust et al., 2021). Additionally, an adequate preliminary reliability was obtained for the instrument as a whole (α = 0.984 and ω = 0.985).

### 3.3. Preliminary analysis

The descriptive analysis of the 50 items of the ESS-P was carried out in two groups: the EFA group with 230 participants and the CFA group with 188 participants. For each item, the mean, standard deviation, skewness index (g_1_), and kurtosis index (g_2_) were provided. The values of the skewness index (g1) for both groups were found within the range of ±2, indicating a symmetrical distribution of the data (Finney and DiStefano, 2006). Most of the values of the kurtosis index (g2) were also found within the range of ±2, indicating a relatively normal distribution of the data. In the EFA group, the item with the highest mean is item 2 with a score of 3, closely followed by item 8 with a score of 2.54. On the other hand, the item with the lowest mean is item 24 with a score of 2.03, closely followed by item 1 with a score of 2.76. In the CFA group, the item with the highest mean is item 17 with a score of 2.37, closely followed by item 18 with a score of 2.36. On the other hand, the item with the lowest mean is item 24 with a score of 1.98, closely followed by item 1 with a score of 2.72 (Table 2).

**Table 2:** Preliminary analysis of the items.

	EFA *n* = 230					CFA *n* = 188			
	Mean	SD	g_1_	g_2_		Mean	SD	g_1_	g_2_
Item 1	2.76	0.77	−0.01	−0.60		2.72	0.88	−0.29	−0.38
Item 2	3.00	0.69	−0.31	−0.02		2.99	0.79	−0.50	−0.10
Item 3	2.65	0.75	0.00	−0.42		2.65	0.84	−0.23	−0.53
Item 4	2.80	0.66	−0.12	−0.11		2.71	0.74	−0.04	−0.41
Item 5	2.68	0.78	0.07	−0.58		2.61	0.80	−0.08	−0.47
Item 6	2.46	0.87	0.00	−0.33		2.47	0.93	−0.04	−0.56
Item 7	2.53	0.77	0.01	−0.42		2.47	0.79	−0.19	−0.17
Item 8	2.54	0.74	−0.07	−0.32		2.51	0.76	−0.04	−0.37
Item 9	2.72	0.74	−0.21	−0.22		2.63	0.81	−0.19	−0.17
Item 10	2.32	0.82	0.03	−0.17		2.31	0.82	0.20	−0.47
Item 11	2.43	0.81	−0.11	−0.08		2.36	0.84	0.14	−0.34
Item 12	2.63	0.76	−0.10	−0.36		2.60	0.87	−0.24	−0.17
Item 13	2.60	0.75	−0.22	0.09		2.57	0.88	−0.18	−0.28
Item 14	2.54	0.74	−0.07	0.03		2.53	0.86	−0.36	−0.13
Item 15	2.58	0.78	−0.14	−0.38		2.48	0.88	−0.12	−0.10
Item 16	2.50	0.80	−0.04	−0.22		2.49	0.83	0.01	−0.31
Item 17	2.43	0.75	−0.08	0.23		2.37	0.82	0.05	−0.01
Item 18	2.40	0.77	−0.05	−0.17		2.36	0.86	−0.02	−0.30
Item 19	2.43	0.73	0.33	−0.21		2.45	0.84	−0.11	−0.10
Item 20	2.43	0.79	−0.10	0.03		2.43	0.82	0.01	0.00
Item 21	2.27	0.78	0.15	−0.44		2.21	0.93	0.06	−0.25
Item 22	2.40	0.81	0.08	−0.51		2.33	0.83	0.07	−0.61
Item 23	2.30	0.85	0.13	−0.23		2.20	0.91	0.03	−0.21
Item 24	2.03	0.88	−0.03	−0.20		1.98	0.95	0.08	−0.28
Item 25	2.29	0.77	0.04	−0.49		2.27	0.89	0.25	−0.70
Item 26	2.18	0.86	−0.10	−0.01		2.15	0.94	0.13	−0.16
Item 27	2.40	0.74	−0.07	0.30		2.48	0.84	0.15	−0.35
Item 28	2.28	0.83	0.21	−0.09		2.30	0.86	0.21	−0.37
Item 29	2.34	0.78	0.14	−0.12		2.37	0.86	0.02	−0.50
Item 30	2.33	0.76	0.09	−0.06		2.31	0.85	0.04	−0.47
Item 31	2.23	0.80	0.14	−0.27		2.17	0.85	0.19	−0.48
Item 32	2.45	0.84	−0.06	−0.41		2.43	0.81	−0.03	−0.27
Item 33	2.50	0.81	−0.16	−0.01		2.44	0.88	0.08	−0.50
Item 34	2.31	0.84	0.07	−0.07		2.24	0.97	−0.10	−0.45
Item 35	2.51	0.79	0.01	−0.17		2.49	0.88	−0.08	−0.52
Item 36	2.48	0.84	−0.12	−0.40		2.53	0.92	−0.17	−0.48
Item 37	2.46	0.81	0.00	−0.28		2.49	0.82	−0.13	0.03
Item 38	2.58	0.75	−0.16	0.04		2.55	0.89	0.00	−0.57
Item 39	2.43	0.75	0.01	−0.37		2.46	0.85	0.01	−0.39
Item 40	2.38	0.77	0.08	−0.40		2.37	0.83	0.14	−0.26
Item 41	2.38	0.77	−0.01	−0.14		2.40	0.82	0.05	−0.29
Item 42	2.36	0.80	0.04	−0.24		2.37	0.86	0.02	−0.47
Item 43	2.42	0.75	0.08	−0.33		2.37	0.83	0.07	−0.31
Item 44	2.37	0.79	0.00	0.05		2.36	0.86	−0.10	−0.32
Item 45	2.35	0.74	0.12	−0.28		2.37	0.86	0.09	−0.20
Item 46	2.57	0.77	0.01	−0.42		2.56	0.85	−0.02	−0.41
Item 47	2.53	0.75	−0.07	−0.33		2.51	0.84	−0.03	−0.33
Item 48	2.58	0.82	0.04	−0.58		2.52	0.86	−0.10	−0.20
Item 49	2.57	0.82	−0.17	−0.27		2.54	0.84	−0.17	−0.31
Item 50	2.46	0.86	−0.30	0.01		2.41	0.88	−0.03	−0.54

EFA, Exploratory Factor Analysis sample; CFA, Confirmatory Factor Analysis sample; M, Mean; SD, Standard Deviation; g_1_, skewness; g_2_, kurtosis.

### 3.4. Preliminary evidence-based internal structure

**Figure 1:**
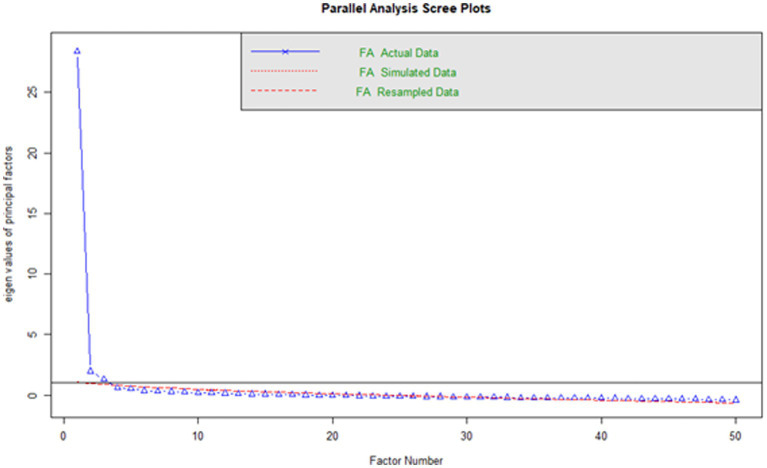
Scree plot.

The sample (*n* = 230) was found to be adequate for conducting the Exploratory Factor Analysis (EFA) through the Kaiser-Meyer-Olkin (KMO) measure of sample adequacy and the Bartlett’s test of sphericity. The KMO was 0.969, indicating an adequate measure of sample adequacy. Additionally, Bartlett’s test was significant (*p* < 0.001). The number of factors was determined through parallel analysis and the scree plot (Figure 1), which suggested the existence of 3 factors. The EFA was carried out using the minimum residuals extraction method with oblimin rotation, retaining only those items with a factor loading greater than or equal to 0.4 and not presenting factorial complexity that could make interpretation difficult. Finally, 39 items organized into a 3-factor structure were obtained (Table 3), which were decided to be named: reading (Factor 1), Oral Communication (Factor 2), and writing (Factor 3).

**Table 3:** Exploratory and confirmatory factor analysis of the items.

	AFE			AFC									Model 1			Model 2			Model 3		
Item				F1	F2	F3	F1	F2	F3	F1	F2	F3	F1	F2	F3
Item1	0.612			0.71			0.69					
Item2	0.716			0.68								
Item3	0.731			0.72			0.71			0.71		
Item4	0.757			0.76			0.75					
Item5	0.672			0.68						0.74		
Item6	0.67			0.72			0.72			0.72		
Item7	0.828			0.85			0.85			0.85		
Item8	0.775			0.83			0.83			0.83		
Item9	0.782			0.78			0.78			0.78		
Item10	0.673			0.8			0.8			0.8		
Item11	0.689			0.84			0.85			0.85		
Item12	0.774			0.82			0.82			0.82		
Item13	0.731			0.85			0.85			0.85		
Item14	0.817			0.86			0.86			0.87		
Item15	0.558			0.81			0.82			0.82		
Item16	0.681			0.81			0.82			0.82		
Item18		0.417			0.82			0.81			0.81	
Item19		0.403			0.83			0.83			0.83	
Item21		0.628			0.83			0.83			0.83	
Item22		0.435			0.77			0.76			0.76	
Item23		0.611			0.82			0.81			0.81	
Item24		0.687			0.83			0.83			0.83	
Item25		0.477			0.81			0.82			0.82	
Item26		0.575			0.83			0.83			0.83	
Item28		0.434			0.81			0.82			0.82	
Item37			0.728			0.87			0.87			0.87
Item38			0.668			0.83			0.83			0.83
Item39			0.518			0.84			0.84			0.84
Item40			0.552			0.82			0.82			0.82
Item41			0.531			0.85			0.85			0.85
Item42			0.445			0.83			0.84			0.84
Item43			0.662			0.86			0.87			0.87
Item44			0.63			0.8			0.81			0.81
Item45			0.697			0.82			0.84			0.84
Item46			0.769			0.82			0.83			0.83
Item47			0.702			0.81			0.82			0.81
Item48			0.88			0.74			0.77			0.77
Item49			0.957			0.85			0.85			0.85
Item50			0.873			0.84			0.84			0.84
				AVE						0.65	0.67	0.7
				F1							0.76	0.85
				F2						0.87		0.85
				F3						0.92	0.92	
				α						0.96	0.95	0.97
				ω						0.96	0.95	0.97

F1, Reading (R); F2, Oral Communication (OC); F3, Writing (W); α, Cronbach’s alpha; ω, McDonald’s Omega; λ, Factor loading; AVE, average variance extracted; below the diagonal, interfactor correlations; above the diagonal, variance shared between factors (AVE > φ^2^).

### 3.5. Internal structure validation

Three confirmatory factor analysis (CFA) models were carried out based on the three factors and 39 items identified in an exploratory factor analysis (EFA), using a sample of 188 participants (*n* = 188). In the first model, adequate fit indices were found: χ^2^ = 1231.460, df = 699, *p* ≤ 0.001, CFI = 0.91, TLI = 0.90, RMSEA = 0.06 (90% CI 0.06–0.07), SRMR = 0.04. However, it was decided to retain only factor loadings (λ) greater than 0.70, leading to the removal of items 2 and 5. The second model also presented adequate fit values: χ^2^ = 1075.910, df = 626, p = <0.001, CFI = 0.92, TLI = 0.91, RMSEA = 0.06 (90% CI 0.06–0.07), SRMR = 0.04. However, by retaining factor loadings (λ) greater than 0.70, item 1 was eliminated. In the third and final model, adequate fit indices were obtained: χ^2^ = 1015.890, df = 591, *p* < 0.001, CFI = 0.92, TLI = 0.92, RMSEA = 0.06 (90% CI 0.06–0.07), SRMR = 0.04, and all factor loadings (λ) were greater than 0.70. Moreover, the average variance extracted (AVE) value was acceptable, being higher than 0.50 for each dimension (AVE > 0.50). However, convergent validity was not adequate, as it was observed that the AVE values were smaller than the shared variance between factors (AVE > φ^2^) in all cases. Despite this, internal consistency was satisfactory, with Cronbach’s alpha and McDonald’s omega coefficients for Reading (α/ω = 0.96), Oral Communication (α/ω = 0.95), and Writing (α/ω = 0.97) showing adequate values.

### 3.6. Second-order model

**Figure 2:**
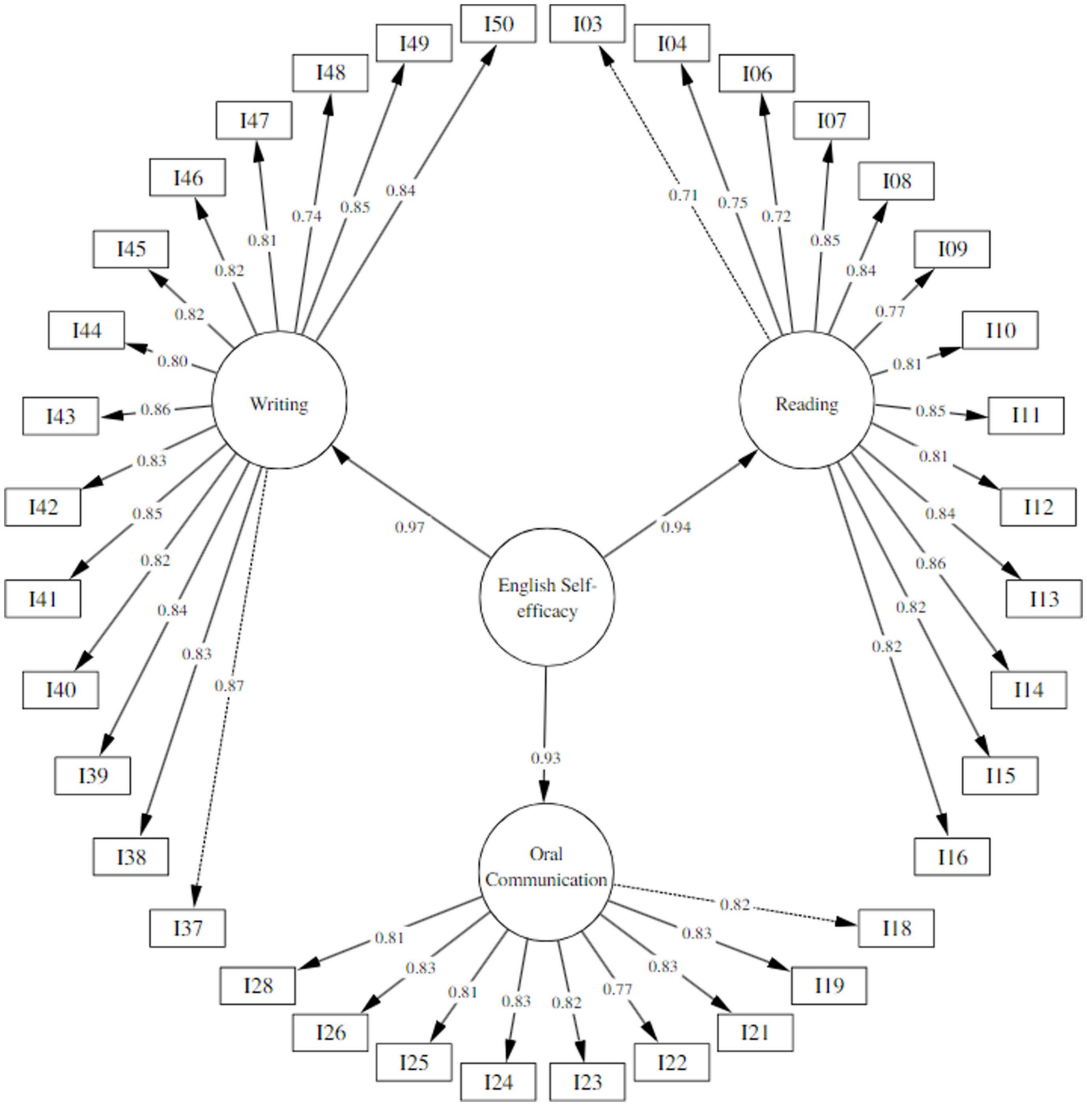
Second order confirmatory factor analysis path diagram.

Because a discriminant validity was not obtained and that the values of the correlations between the factors were greater than 0.80, which would indicate that the dimensions belong to a second-order factor, so a model was made (Mason and Perreault, 1991; Schwarz et al., 2014) with 3 first-order and 1 second-order factors, similar to several models presented in previous studies for the same construct (Wang et al., 2014; Ngoc Truong and Wang, 2019; Chauvin et al., 2020), obtaining adequate adjustment indices χ^2^ = 1021.61, gl = 591, *p* < 0.001, CFI = 0.92, TLI = 0.92, RMSEA = 0.06 [IC 90% = 0.05–0.07] y SRMR = 0.04. Regarding this model, the internal consistency coefficients Cronbach alpha and McDonald’s Omega for the dimensions Reading (α/ω = 0.96), Oral Communication (α/ω = 0.95) y Writing (α/ω = 0.97) as well as for the full scale (α/ω = 0.98), showed adequate values (Figure 2).

### 3.7. Factor invariance by sex

The factor invariance between sexes was evaluated. Table 4 shows evidence of strict invariance according to the Δ CFI criterion. When adding the equal means restriction, the model fit did not worsen significantly, suggesting that the latent means are similar for both sexes. Therefore, models M1, M2, M3, and M4 meet the expected criteria and confirm the factor invariance of the ESS-P. This allows for comparison of different measures in sex groups.

**Table 4:** Factor invariance by sex.

	χ^2^	df	RMSEA	*p*	SRMR	TLI	CFI	∆CFI
M1	1938.441	1,182	0.06	<0.001	0.04	0.93	0.93	
M2	1974.166	1,215	0.06	<0.001	0.05	0.93	0.93	<0.001
M3	2014.413	1,248	0.05	<0.001	0.05	0.93	0.93	0.001
M4	2041.688	1,284	0.05	<0.001	0.05	0.93	0.93	−0.001

M1, configural; M2, Metric; M3, Scalar; M4, Strict; χ2, Chi-square; df, degrees of freedom; RMSEA, Root Mean Square Error of Approximation; SRMR, Standardized Root Mean-Square; TLI, Tucker-Lewis Index; CFI, Comparative Fit Index; ΔCFI, Comparative Fit Index difference.

### 3.8. Validation based on relationship with other variables

**Figure 3:**
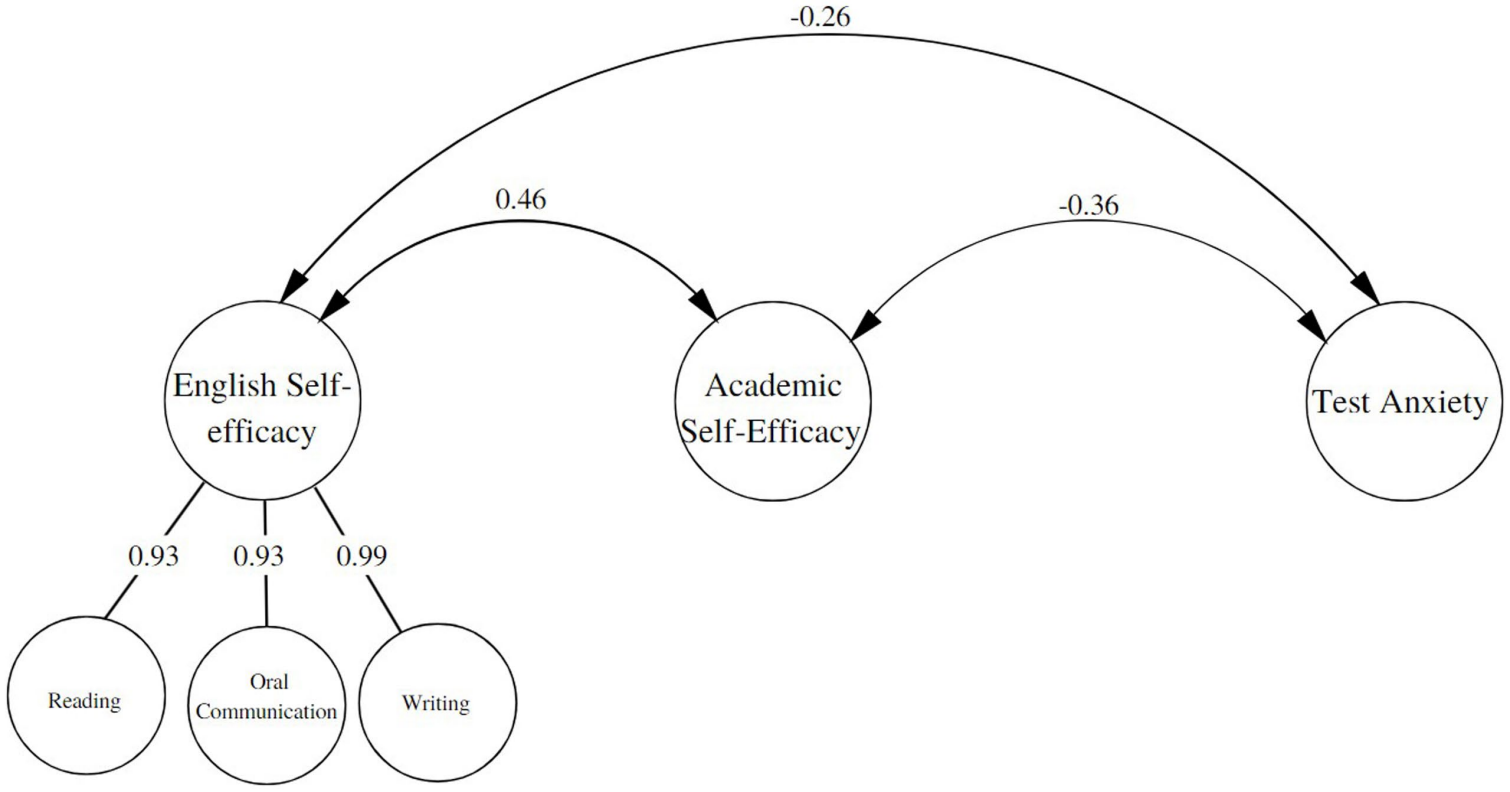
Structural model of structural equations for convergent validity of the ESS-P.

Based on a literature review, we proposed a model to assess the convergence of the ESS-P with other measures. For this purpose, a structural equation modeling (SEM) approach was used to examine the latent relationships between the dimensions of the ESS-P (Reading, Oral Communication, and Writing) and the variables Academic self-efficacy and Test Anxiety. The structural model showed adequate fit indices: χ^2^ = 1115.750, gl = 661, *p* = <0.001 CFI = 0.92, TLI = 0.91, RMSEA = 0.06 (90% CI 0.05–0.07), SRMR = 0.04. Furthermore, the measurement models were appropriately represented by their items, as shown in Figure 3. The results revealed that the dimensions of the ESS-P (Reading, Oral Communication, and Writing) are positively related to academic self-efficacy and negatively related to test anxiety. These relationships suggest that as proficiency in English skills increases, academic self-efficacy also increases, and test anxiety decreases. Taking these results into account, it can be concluded that the ESS-P demonstrates strong evidence of convergent validity, indicating that this assessment tool is consistent with other relevant measures in the academic and psychological domains. This convergence supports the usefulness of the ESS-P as a valid instrument for measuring English skills in students and their relationship with academic self-efficacy and test anxiety.

## 4. Discussion

Self-efficacy refers to an individual’s belief in their ability to perform a task or achieve a goal. Self-efficacy in second language learning, such as English, plays an important role in student success and motivation. Thus, in Peru and other Spanish-speaking countries, appropriate instruments are needed to measure English self-efficacy, considering dimensions such as: reading, writing and oral communication. The aim of this study was to develop an English Self-Efficacy Scale (ESS-P) and provide the first evidence of validity, reliability, and invariance according to sex in Peruvian university students.

The evidence based on the content was carried out in two phases. First, a literature review was conducted to provide information about the scales developed and related ideas about the concept of English self-efficacy. Second, the representativeness and relevance of the items were verified by 5 experts, as relevance and representativeness are important when developing a scale as they provide quality and coherence of the items according to the theoretical aspects of the scale being developed (Clark and Watson, 1995; Eignor, 2013). A pilot test was also conducted to qualitatively verify the participants’ understanding of the items, obtaining adequate discrimination indices for each of them (>0.2; Rust et al., 2021).

Evidence based on the internal structure of the ESS-P was carried out in two phases. In the first phase, an Exploratory Factor Analysis (EFA) was used which showed the existence of three proposed factors (Reading, Writing, and Oral Communication). Then, a Confirmatory Factor Analysis (CFA) based on the structure of the EFA was carried out, which showed that the goodness-of-fit indices were adequate. In this way, the theoretical proposal of three dimensions to evaluate English self-efficacy was empirically tested, which are similar to previous proposals (Wang et al., 2013, 2014; Chauvin et al., 2020; Kim et al., 2021). The factors were named: (a) reading, which refers to the belief in the ability to understand written texts in English; (b) writing, refers to the confidence in the ability to produce written texts in English with meaning and coherence (Wang et al., 2013, 2014; Kim et al., 2021); and (c) oral communication which refers to the confidence in the ability to communicate effectively through spoken language (Chauvin et al., 2020). Additionally, the factor loads were adequate, greater than 0.50, indicating a robust factor structure, as the items are influenced in a homogeneous and strong manner by the latent variable. This means that each aspect of the construct of English self-efficacy is adequate and relevant to the construct being studied, and allows subjects with high and low English self-efficacy to be differentiated (Kline, 2016).

Reliability of the ESS-P was assessed in terms of each dimension using Cronbach’s Alpha and McDonald’s Omega (ω) coefficients, which indicated that the ESS-P is internally consistent with values greater than 0.70 (Raykov and Hancock, 2005). Therefore, the ESS-P is considered a reliable tool.

In terms of convergent validity, which seeks to ensure that the items of a variable reflect the corresponding factor, it is measured through the positive correlation between the indicators of the same variable. Average Variance Extracted (AVE) is a common indicator for measuring convergent validity and is considered acceptable if it is greater than 0.5 (Fornell and Bookstein, 1982). An AVE less than 0.5 indicates that the explained variance is less than the error variance (Chin, 1998). In the present three-dimensional model, the AVE exceeded the threshold with a value greater than 0.50, which is considered acceptable. Although convergent validity was not adequate, as the AVE values were lower than the shared variance between factors (AVE > φ2) in all cases, and the correlations between factors were greater than 0.80, a second-order model with 3 factors was proposed (Mason and Perreault, 1991; Schwarz et al., 2014), similar to previous studies (Wang et al., 2014; Ngoc Truong and Wang, 2019; Chauvin et al., 2020).

The factor invariance analysis in relation to the ESS-P and sex has shown the stability of the thresholds, factor loadings, intercepts, and residuals across groups. This indicates that the items measure the latent variable in the same way for both men and women (Brown, 2015). Therefore, it can be stated that differences in the scores of men and women are due to differences in the latent trait and not a bias in the measurement instrument. These results are important because they will allow for future studies on self-efficacy based on sex and provide useful information for its application.

The evidence based on the relationship with other variables showed that the ESS-P is correlated with academic variables such as self-efficacy and anxiety, showing positive correlations of the scores of English self-efficacy with academic self-efficacy and negative correlation with anxiety toward the exams, which would be supported by previous studies (Preiss et al., 2006; Wang et al., 2018). Similarly, the correlation between the ESS-P and the academic level of the participants was verified, because there is evidence of the association of self-efficacy for English and academic achievement (Kitikanan and Sasimonton, 2017).

### 4.1. Implications

The English Self-Efficacy Scale (ESS-P) provides a valuable resource for educators and professionals to assess students’ self-efficacy beliefs in reading, writing, and oral communication skills in English. This information can guide professionals in identifying areas where students may need additional support, designing interventions to improve their self-efficacy, and ultimately, their English skills. Furthermore, by taking into account gender differences in self-efficacy, professionals can tailor their strategies to address the specific needs of men and women in learning English. Additionally, policymakers can use the findings of these studies to inform the development of programs and educational policies that promote English learning in Spanish-speaking communities. This may include creating curricula and educational materials that cater to the specific needs of self-efficacy in reading, writing, and oral communication, as well as implementing teacher training programs that teach them how to assess and foster self-efficacy in their students. Also, empirical evidence supports the three-factor structure of the ESS-P. This can serve as a foundation for future research that delves deeper into self-efficacy and its relationship with English learning in different contexts and populations.

### 4.2. Limitations

The sample for this study focused on Peruvian university students, which limits the generalization of the results to other contexts and populations. Future research could expand the sample to include students from different educational levels, age groups, and cultural contexts, allowing for the evaluation of the applicability and validity of the ESS-P in various situations. Moreover, the cross-sectional design of the study does not allow for establishing causal relationships between English self-efficacy and other variables, such as academic performance or life satisfaction. Future research could employ longitudinal or experimental designs to examine the effects of English self-efficacy on learning and success over time. Additionally, a test–retest analysis was not conducted to assess the temporal stability of the ESS-P, thus including this evaluation in future studies is recommended. Although acceptable convergent validity was identified through AVE, it is suggested to analyze the items using an Item Response Theory model. While the sample was heterogeneous, with different levels of English proficiency, these differences did not significantly affect the model’s goodness of fit. However, future studies may need to examine these differences among populations, as this was not the main objective of the present study. Furthermore, researchers could explore the interaction of self-efficacy with other variables, such as motivation, learning strategies, and social support, to gain a more comprehensive understanding of the factors contributing to English learning.

## 5. Conclusion

The ESS-P is a valid and reliable instrument that assesses three dimensions of English self-efficacy: reading, writing, and oral communication for measuring English in Peruvian university students. Moreover, it has been demonstrated that the scale is invariant across gender, allowing for comparisons between men and women. The development of the scale contributes to the field of research in teaching and learning English as a second language in Spanish-speaking contexts. The scale can be a useful tool for researchers and practitioners seeking to understand and improve English self-efficacy and, ultimately, student performance and motivation. The implications of these findings can inform educational practices and policies, focusing on promoting English self-efficacy and addressing gender differences.

## Data availability statement

The raw data supporting the conclusions of this article will be made available by the authors, without undue reservation.

## Ethics statement

The research protocol was approved by the Research Ethics Committee of the Universidad Peruana Unión with reference number CE-EPG-0000119. The patients/participants provided their written informed consent to participate in this study.

## Author contributions

MM-T, DC-A, and WM-G participated in the conceptualization of the idea. LS-S, OR-L, and SM-G were in charge of the methodology and software. WM-G, MM-T, FS-S, and DC-A were responsible for validation, formal analysis, and research. Data curation and resources were commissioned by WM-G, LS-S, SM-G, FS-S, and OR-L. The writing of the first draft, review and editing, visualization and supervision were carried out by MM-T, WM-G, DC-A, LS-S, SM-G, and OR-L. All authors contributed to the article and approved the submitted version.

## Conflict of interest

The authors declare that the research was conducted in the absence of any commercial or financial relationships that could be construed as a potential conflict of interest.

## Publisher’s note

All claims expressed in this article are solely those of the authors and do not necessarily represent those of their affiliated organizations, or those of the publisher, the editors and the reviewers. Any product that may be evaluated in this article, or claim that may be made by its manufacturer, is not guaranteed or endorsed by the publisher.

## Supplementary material

The Supplementary material for this article can be found online at: https://www.frontiersin.org/articles/10.3389/fpsyg.2023.1187342/full#supplementary-material
