# Supplementary material for: Psychometric properties and invariance of an English self-efficacy scale for university students in Peru
Source: Front Psychol. 2023 Jun 15;14:1187342. doi: 10.3389/fpsyg.2023.1187342 (PMC10310963; doi:10.3389/fpsyg.2023.1187342)
Supplement: Supplementary file 1 [file Table_1.docx]

**Annex 1**

**Escala de Autoeficacia en inglés**

Instrucciones

Las personas difieren en la confianza que tienen en hacer diversas actividades. En relación con el uso del idioma inglés, califique qué tan seguro(a) está de que puede realizar cada una de las siguientes actividades al indicar una probabilidad de éxito de 0 (nada probable) a 4 (certeza completa).

Recuerde que su sinceridad es muy importante, no hay respuestas buenas ni malas, asegúrese de contestar todas.

| **0** | **1** | **2** | **3** | **4** |
| --- | --- | --- | --- | --- |
| No puedo hacerlo en lo absoluto | No puedo hacerlo | Relativamente seguro de poder hacerlo | Puedo hacerlo | Totalmente seguro de poder hacerlo |

| Ítems |  | | | | |
| --- | --- | --- | --- | --- | --- |
| **Reading** | | | | | |
| A partir de una inspección rápida del texto en inglés, predecir de qué tratará. | 0 | 1 | 2 | 3 | 4 |
| Reconocer las palabras presentes en el texto. | 0 | 1 | 2 | 3 | 4 |
| Identificar un tipo de texto escrito en inglés (ensayo, nota periodística, infografía, revista, etc.). | 0 | 1 | 2 | 3 | 4 |
| Comprender cuál es el propósito del autor de un texto escrito en inglés. | 0 | 1 | 2 | 3 | 4 |
| Identificar cuál es el mensaje específico de cada oración y párrafo de un texto escrito en inglés. | 0 | 1 | 2 | 3 | 4 |
| Reconocer el significado de la mayoría de las palabras de un texto escrito en inglés. | 0 | 1 | 2 | 3 | 4 |
| Identificar las ideas principales que el autor no menciona de forma explícita en un texto escrito en inglés. | 0 | 1 | 2 | 3 | 4 |
| Construir la idea central de todo un texto escrito en inglés, a partir de las ideas principales de cada párrafo. | 0 | 1 | 2 | 3 | 4 |
| Extraer información específica de un texto escrito en inglés, para responder las interrogantes que se presenten. | 0 | 1 | 2 | 3 | 4 |
| Localizar información específica de un texto escrito en inglés, con el fin de verificar datos relevantes. | 0 | 1 | 2 | 3 | 4 |
| Reconocer el mensaje que el autor desea trasmitir, mediante su texto escrito en inglés. | 0 | 1 | 2 | 3 | 4 |
| Expresar si se está de acuerdo o no con lo que el autor plantea en un texto escrito en inglés. | 0 | 1 | 2 | 3 | 4 |
| Aplicar la información de un texto escrito en inglés, a tareas académicas o profesionales específicas. | 0 | 1 | 2 | 3 | 4 |
| **Oral communication** | | | | | |
| Preguntar en inglés al interlocutor cuando se necesita información sobre un tema específico. | 0 | 1 | 2 | 3 | 4 |
| Lograr que la otra persona comprenda un mensaje hablado en inglés. | 0 | 1 | 2 | 3 | 4 |
| Dialogar sobre un tema en inglés, dando a conocer al interlocutor los propios puntos de vista. | 0 | 1 | 2 | 3 | 4 |
| Utilizar el idioma inglés para comunicarse en diferentes situaciones de la vida cotidiana. | 0 | 1 | 2 | 3 | 4 |
| Hablar en inglés de manera espontánea. | 0 | 1 | 2 | 3 | 4 |
| Hablar en inglés de forma fluida. | 0 | 1 | 2 | 3 | 4 |
| Hablar en inglés con una entonación adecuada. | 0 | 1 | 2 | 3 | 4 |
| Utilizar la velocidad necesaria al hablar en inglés, según las circunstancias. | 0 | 1 | 2 | 3 | 4 |
| Exponer ideas en inglés, utilizando correctamente las normas y reglas gramaticales. | 0 | 1 | 2 | 3 | 4 |
| **Writing** | | | | | |
| Tener un propósito claro al escribir en inglés. | 0 | 1 | 2 | 3 | 4 |
| Investigar o informarse sobre el tema que se va a escribir en inglés. | 0 | 1 | 2 | 3 | 4 |
| Escribir en inglés de acuerdo al destinatario (familiar, directivo, docente, empleador, etc.). | 0 | 1 | 2 | 3 | 4 |
| Planificar el tipo de texto y los recursos a utilizar, para escribir en inglés. | 0 | 1 | 2 | 3 | 4 |
| Utilizar lenguaje formal o informal en el texto que se escribe en inglés, según el público objetivo. | 0 | 1 | 2 | 3 | 4 |
| Utilizar un vocabulario variado, al escribir en inglés. | 0 | 1 | 2 | 3 | 4 |
| Presentar de forma ordenada y comprensible las ideas en el texto, al escribir en inglés. | 0 | 1 | 2 | 3 | 4 |
| Evitar las repeticiones innecesarias, al escribir en inglés. | 0 | 1 | 2 | 3 | 4 |
| Utilizar conectores variados y apropiados al escribir en inglés, para enlazar ideas. | 0 | 1 | 2 | 3 | 4 |
| Hacer buen uso de los signos de puntuación, al escribir en inglés. | 0 | 1 | 2 | 3 | 4 |
| Escribir correctamente las palabras en inglés, haciendo uso de una ortografía adecuada. | 0 | 1 | 2 | 3 | 4 |
| Elaborar el primer borrador de un texto en inglés. | 0 | 1 | 2 | 3 | 4 |
| Revisar el texto en inglés al culminar de escribirlo, para verificar si se cumplió con el objetivo propuesto. | 0 | 1 | 2 | 3 | 4 |
| Corregir los errores de redacción de un texto en inglés y presentar la versión final. | 0 | 1 | 2 | 3 | 4 |
