# Supplementary material for: Psychometric properties and invariance of an English self-efficacy scale for university students in Peru
Source: Front Psychol. 2023 Jun 15;14:1187342. doi: 10.3389/fpsyg.2023.1187342 (PMC10310963; doi:10.3389/fpsyg.2023.1187342)
Supplement: Supplementary file 2 [file Table_2.docx]

**Appendix 1**

Self-Efficacy Scale in English

Instructions

People differ in how confident they are in doing various activities. Regarding the use of the English language, please rate how confident you are that you can perform each of the following activities by indicating a probability of success from 0 (not at all likely) to 4 (completely certain).

Remember that your honesty is very important, there are no right or wrong answers, so be sure to answer all of them.

| **0** | **1** | **2** | **3** | **4** |
| --- | --- | --- | --- | --- |
| I cannot do it at all | I cannot do it. | Relatively sure I can do it | I can do it | I am totally sure I can do it |

| Ítems |  | | | | |
| --- | --- | --- | --- | --- | --- |
| **Reading** | | | | | |
| From a quick inspection of an English text, predict what it will be about. | 0 | 1 | 2 | 3 | 4 |
| Recognize the words present in a text. | 0 | 1 | 2 | 3 | 4 |
| Identify a type of text written in English (essay, journalistic note, infographic, magazine, etc.). | 0 | 1 | 2 | 3 | 4 |
| Understand the author's purpose of a text written in English. | 0 | 1 | 2 | 3 | 4 |
| Identify the specific message of each sentence and paragraph of a text written in English. | 0 | 1 | 2 | 3 | 4 |
| Recognize the meaning of most words in a text written in English. | 0 | 1 | 2 | 3 | 4 |
| Identify the main ideas that the author does not explicitly mention in a text written in English. | 0 | 1 | 2 | 3 | 4 |
| Construct the central idea of a whole text written in English, from the main ideas of each paragraph. | 0 | 1 | 2 | 3 | 4 |
| Extract specific information from a text written in English to answer the questions that are presented. | 0 | 1 | 2 | 3 | 4 |
| Locate specific information from a written text in English in order to verify relevant data. | 0 | 1 | 2 | 3 | 4 |
| Recognize the message that the author wants to convey through his or her written text in English. | 0 | 1 | 2 | 3 | 4 |
| Express whether you agree or disagree with what the author is saying in a text written in English. | 0 | 1 | 2 | 3 | 4 |
| Apply information from a text written in English to specific academic or professional tasks. | 0 | 1 | 2 | 3 | 4 |
| **Oral communication** | | | | | |
| Ask the interlocutor in English when information is needed on a specific topic. | 0 | 1 | 2 | 3 | 4 |
| Get the other person to understand a message spoken in English. | 0 | 1 | 2 | 3 | 4 |
| Talk about a topic in English, letting the interlocutor know your own points of view. | 0 | 1 | 2 | 3 | 4 |
| Use the English language to communicate in different situations of everyday life. | 0 | 1 | 2 | 3 | 4 |
| Speak in English spontaneously. | 0 | 1 | 2 | 3 | 4 |
| Speak English fluently. | 0 | 1 | 2 | 3 | 4 |
| Speak in English with appropriate intonation. | 0 | 1 | 2 | 3 | 4 |
| Use the necessary speed to speak in English, depending on the circumstances. | 0 | 1 | 2 | 3 | 4 |
| Present ideas in English, using grammatical rules and norms correctly. | 0 | 1 | 2 | 3 | 4 |
| **Writing** | | | | | |
| Have a clear purpose when writing in English. | 0 | 1 | 2 | 3 | 4 |
| Research or learn about the topic to be written in English. | 0 | 1 | 2 | 3 | 4 |
| Write in English according to the recipient (family member, manager, teacher, employer, etc.). | 0 | 1 | 2 | 3 | 4 |
| Plan the type of text and the resources to be used, to write in English. | 0 | 1 | 2 | 3 | 4 |
| Use formal or informal language in the text written in English, depending on the target audience. | 0 | 1 | 2 | 3 | 4 |
| Use varied vocabulary when writing in English. | 0 | 1 | 2 | 3 | 4 |
| Present the ideas in the text in an orderly and understandable way, when writing in English. | 0 | 1 | 2 | 3 | 4 |
| Avoid unnecessary repetition when writing in English. | 0 | 1 | 2 | 3 | 4 |
| Use varied and appropriate connectors when writing in English to link ideas. | 0 | 1 | 2 | 3 | 4 |
| Make good use of punctuation marks when writing in English. | 0 | 1 | 2 | 3 | 4 |
| Write English words correctly, using proper spelling. | 0 | 1 | 2 | 3 | 4 |
| Write the first draft of a text in English. | 0 | 1 | 2 | 3 | 4 |
| Review the text in English at the end of writing it, to verify if the proposed objective was met. | 0 | 1 | 2 | 3 | 4 |
| Correct the writing errors of a text in English and present the final version. | 0 | 1 | 2 | 3 | 4 |
